# Effectiveness of Mentorship Using Cognitive Behavior Therapy to Reduce Burnout and Turnover among Nurses: Intervention Impact on Mentees

**DOI:** 10.3390/nursrep14020077

**Published:** 2024-04-23

**Authors:** Takashi Ohue, Masaru Menta

**Affiliations:** 1 Department of Nursing, Faculty of Nursing, Hyogo University, 2301, Sinzaike, Kakogawa 675-0195, Japan; 2 Department of Clinical Psychology, Faculty of Education, Bukkyo University, Kyoto 604-8418, Japan; menta@bukkyo-u.ac.jp

**Keywords:** nurse, burnout, cognitive behavioral therapy, mentorship program

## Abstract

Objective: Mentoring programs can improve nurses’ mental health. This study examined the effects of a staff training program based on cognitive behavior therapy for burnout in which mentors provided intervention to their mentees. Methods: The principal investigator served as a facilitator and conducted staff training in cognitive behavior therapy. An original cognitive behavior therapy manual was presented to trained nurses (mentors), and lectures were provided on using the manual, ways of implementing cognitive behavior therapy, and other important points. The study participants included 35 mid-career nurses (mentors) and 34 young nurses in their first to third year (mentees) working in acute care hospitals. Groups of five mentees were formed in which two mentors provided cognitive behavior therapy based on the manual. Changes in mentees’ stress, burnout, and turnover intention at pre-intervention, post-intervention, and follow-up (3 months after the intervention) were objectively evaluated using an evaluation index. Results: The intervention significantly reduced the following evaluation indicators: total strain, conflict with other nursing staff, nursing role conflict, qualitative workload, quantitative workload, conflict with patients, problem avoidance due to irrational beliefs, escape-avoidance, emotional exhaustion of burnout, desire to change hospitals or departments, and turnover intention. Conclusion: Implementation of cognitive behavior therapy by mentors effectively reduced mentees’ stress, burnout, and turnover.

## 1. Introduction

The COVID-19 pandemic has posed unprecedented challenges to healthcare systems worldwide, with nurses playing a pivotal role in the frontline response. This literature review examines the profound impact of the pandemic on the mental health of nurses, exploring the diverse stressors, coping mechanisms, and support strategies identified in empirical research and scholarly literature. Drawing upon a comprehensive review of relevant studies, this paper elucidates the multifaceted nature of mental health challenges faced by nurses during the COVID-19 crisis.

The mental health implications of the COVID-19 pandemic on nurses are manyfold and profound. Studies have consistently highlighted elevated levels of anxiety, stress, and psychological distress among nurses, stemming from factors such as increased workload, fear of infection, inadequate personal protective equipment (PPE), and moral dilemmas in patient care [1]. The prolonged exposure to traumatic events and the relentless pressure of caring for critically ill patients have exacerbated burnout and compassion fatigue among nurses, further compromising their mental well-being [2,3].

In response to these challenges, nurses employ a variety of coping mechanisms to mitigate the adverse effects on their mental health. Social support networks, both within the healthcare setting and from family and peers, have been identified as crucial protective factors against psychological distress [4]. Peer support programs, online forums, and debriefing sessions offer avenues for emotional expression and solidarity, facilitating resilience and coping among nurses facing unprecedented stressors [5].

Organizational support and resources play a pivotal role in promoting nurses’ mental health and well-being during the pandemic. Adequate staffing, access to mental health services, and clear communication channels are essential components of a supportive work environment [6]. Interventions such as stress management training, mindfulness-based programs, and psychological debriefing sessions have shown promise in enhancing nurses’ coping skills and reducing psychological distress [7].

Thus, the importance of mental health support for nurses has been highlighted due to the impact of the novel coronavirus. In the financial year of 2022, the turnover rate of nursing staff increased to 11.6% among full-time nurses (increased by 1.0 points from the previous year), 10.3% among new graduates (increased by 2.0 points), and 16.8% among graduates (increased by 1.9 points) [8]. The term “2025 problem” is being used to denote various problems that are likely to occur when the baby-boom generation (born between 1947 and 1949), which is estimated to be approximately 8 million people in Japan, reaches the age of 75 years or above and enters their later stages of life [9]. Baby boomers encompass a large proportion of the total population in Japan and have been a major influence on society. Furthermore, the nursing workforce turnover is another major issue in today’s society in Japan. Nurse turnover is affected by work overload and burnout. Burnout is commonly defined as a state of emotional, mental, and physical exhaustion caused by prolonged stress or frustration, particularly in the workplace [10]. Numerous studies have highlighted its adverse effects on individuals, including decreased job satisfaction, increased absenteeism, and compromised quality of care [11,12]. According to the “Status of Workers’ Compensation for Brain and Heart Diseases and Mental Disorders” published by the Ministry of Health, Labor, and Welfare (FY2014), medical and welfare professions, including nursing, are reported to have the highest number of workers’ compensation claims for mental disorders [13]. Specifically, mental health problems are common among nurses in their 20s. Therefore, support systems are considered crucial for the younger generation. Cognitive behavioral therapy (CBT) is one of the intervention methods for nurse burnout. CBT is a structured therapeutic approach that targets dysfunctional thoughts and behaviors, aiming to modify them to alleviate psychological distress [14]. In the context of burnout, CBT interventions have shown promise in enhancing coping strategies, promoting resilience, and reducing symptoms of burnout [15,16]. These interventions often incorporate techniques such as cognitive restructuring, problem-solving skills training, and relaxation exercises.

In addition, In Japan, working hours have been regarded as a problem, and efforts have been made to establish a work–life balance in the nursing profession. However, the number of people with mental health problems has not decreased, highlighting the need for a new system. The preceptor system was introduced in Japan in the late 1980s. Its purpose was to alleviate the reality shock experienced by new graduate nurses, promote the growth of preceptors, ensure consistency in teaching new graduate nurses, strengthen practical skills, enhance their thinking and judgment skills, and integrate their technical skills. However, in the preceptor system, owing to the gap in the expectations and values of the preceptors and new graduate nurses, new graduate nurses do not always utilize their preceptors as supporters for reducing the effects of the reality shock experienced shortly after employment [17]. Therefore, a mentor system could be considered as a new support system. The mentoring system is an individualized support program for junior nurses (mentees) provided by senior nurses (mentors) with extensive knowledge and professional experience in hospitals. Mentorship programs involve pairing individuals with experienced mentors who provide guidance, support, and professional development opportunities [18]. Research indicates that mentorship can serve as a protective factor against burnout by offering emotional support, career guidance, and role modeling [19,20]. Mentorship programs have been particularly beneficial for early-career professionals navigating challenging work environments.

Integrating CBT interventions with mentorship programs presents a promising approach to addressing burnout comprehensively. By combining cognitive restructuring techniques with mentor support, individuals can develop adaptive coping strategies while receiving personalized guidance and encouragement. Future research should focus on evaluating the effectiveness of integrated interventions across diverse populations and professions, considering factors such as cultural relevance, organizational support, and long-term sustainability.

Ohue and Menta [21] implemented a cognitive behavioral therapy program for mentors, reporting improvements in mentors’ knowledge of cognitive behavioral therapy and reductions in their own mental health issues. There have been no reports of studies where mentors implemented cognitive behavioral therapy for mentees.

In this paper, mid-career nurses trained in the burnout-focused cognitive behavior therapy (CBT) program developed by Ohue et al. served as mentors and administered CBT to their mentees [22].

## 2. Materials and Methods

### 2.1. Study Design

**Figure 1:**
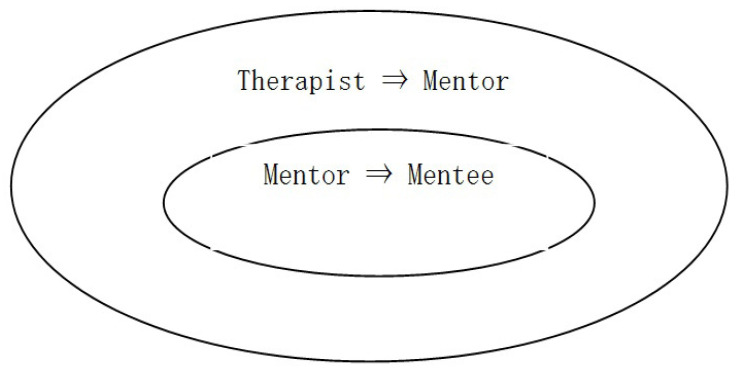
Mentor intervention program for mentees.

The research design involves an intervention study without a control group. The present study used a mentoring system that had a meta-treatment structure and employed a mediated treatment method. The therapist provided CBT to the mentors (mid-level nurses), who then provided CBT to their mentees (Figure 1).

Mid-career nurses with 5–15 years of clinical experience were selected as mentors and received staff training. The inclusion criteria were previous experience as a preceptor, currently in a non-managerial position, and a different department than the mentee. Following selection, the therapist provided staff training to the mentors (mid-level nurses) in a lecture format based on a CBT program for burnout. The training included three 90 min sessions held once a week. Ohue and Menda examined the effectiveness of cognitive behavioral therapy for mentors. According to this report, the mentors were given three cognitive behavioral therapy programs, and it was confirmed that the mentors’ knowledge and skills regarding cognitive behavioral therapy, as well as their listening skills, improved. This program consisted of the same program as the mentee’s and consisted of an experiential learning program for cognitive behavioral therapy [21]. The content included information on using the originally developed CBT manual, ways to implement CBT, and points to be considered.

The author was the therapist for this study. The author is a licensed nurse and certified psychologist and has experience in cognitive behavioral therapy. The second author also served as a supervisor regarding cognitive behavioral therapy.

Thereafter, three CBT programs were conducted with 34 nurses (mentees) with one to three years of clinical experience in CBT. Table 1 presents a summary of the program content for each session. Groups of approximately five mentees were formed, and two mentors intervened as CBT providers based on the manual. The program included three 90 min sessions held once a week. Each program comprised psychoeducational intervention and group work. The first session provided an intervention on stress, burnout, and associated factors among nurses, in which the participants discussed their perceived stressors while performing their current duties. The second session provided an intervention using cognitive restructuring (the relationship between thinking and stress). The third session was a group session that included training and discussion about problem-solving skills, which is effective in reducing burnout.

This study chose a 2-step training model involving therapist–mentors–mentees to ensure comprehensive training and support for the effective implementation of cognitive behavioral therapy (CBT) among mentees. This study refers to the parent training model of Menda et al. [23]. This parent training has a dual structure in which parents provide behavioral therapy intervention to the behavior of children with developmental disabilities. Therapists provide parent training to parents, and parents intervene with their own children. By adopting this model, therapy becomes possible in everyday life.

### 2.2. Evaluation Indicators

(a) Burnout: The Maslach Burnout Inventory (MBI) Japanese version was used [24]. This is a scale introduced by Maslach et al. [25] and modified by Tao et al. [25], consisting of 3 factors: “emotional exhaustion”, “depersonalization” and “personal accomplishment”. Its reliability and validity have been confirmed. It contains a total of 17 items evaluated by five levels from “always present” to “absent”. The higher the scores for “emotional exhaustion” and “depersonalization”, and the lower the scores for “personal accomplishment”, the more likely the participants develop burnout.

(b) Intention to leave the job: According to the categories introduced by Tsuchie et al. [26], presence or absence of Intention to leave the job including “Wants to quit working as a nurse”, “wants to switch hospitals or departments” and “wants to continue working as a nurse” are evaluated at 5 levels from “always present” to “absent”. The higher the scores, the stronger the participants’ will.

(c) Irrational belief of nurses: The scale introduced by Ohue et al. was used [27]. Reliability and validity have been confirmed. The scale consists of 7 factors: “Patient belief”, “Self-expectation”, “problem avoidance”, “self-control”, “logical criticism”, “helplessness” and “dependence” with 28 items. Evaluations were made at 5 levels from “totally agree” to “do not agree at all”; the higher the scores, the more intensively the participants had irrational beliefs. Only the items of the factors “problem avoidance”, “helplessness” and “dependence”, which had been associated with burnout according to the results of the study by Ohue et al. [28], were used.

(d) Automatic thoughts: “the ATQ shorter form” introduced by Ohue et al. was used [29]. Reliability and validity had been confirmed. This scale consists of “Automatic Thoughts Questionnaire-Revised” (hereinafter, ATQ-R) introduced by Kodama et al. [30] with a total of 18 items: 6 items of “negative evaluation of the future”, 6 items of “self-blame”, and 6 items “positive thinking”. Evaluations were made in 5 levels from “totally agree” to “not agree at all”, indicating that the higher the scores, the more intensively the participants have the subscale automatic thoughts.

(e) Coping: “the coping scale” introduced by Ozeki was used for evaluations of coping [31]. This scale is a simplified version of a coping scale introduced by Sakata [32], consisting of a total of 14 items: 5 items of “problem-focused coping”, 3 items of “emotion-focused coping”, and 6 items of “escape-avoidance coping”. Reliability and validity had been confirmed. Evaluations were made in 4 levels from “do not at all” to “always do”, and the scores for 3 subscales could be obtained. The higher the scores, the more the participants had coping behaviors”.

(f) Measurement of stressors: Higashiguchi’s Nursing Job Stressor Scale (NJSS) was used [33]. The NJSS consists of 33 questions that describe potential stressful situations for nurses divided into the following 7 subscales: “conflict with other nursing staff”, “nursing role conflict”, “conflict with physicians/autonomy” “dealing with death and dying”, “qualitative workload”, “quantitative workload”, and “conflict with patients”. The higher the score is, the greater the burden of the stressor is. Only the items of “conflict with other nursing staff”, “nursing role conflict”, “qualitative workload”, “quantitative workload”, and “conflict with patients” were used. (a)–(f) were measured before intervention, after intervention and 3 months later.

### 2.3. Procedures

(a) Ten hospitals were randomly selected from hospitals in Japan.
(b) After the ethics review was approved at each hospital, documents related to the purpose of the research were sent in writing and verbally to the nursing director of each hospital.
(c) Three hospitals for which consent could be obtained were targeted for the study.
(d) After receiving approval from the nursing director, we contacted the nursing department of each hospital and asked the head nurse of each ward to coordinate the work of the targeted mentors and mentees in order to conduct group cognitive behavioral therapy.
(e) The nurse who will serve as a mentor must have previously undergone Ohue and Menda’s cognitive behavioral therapy program [21].
(f) Nurses serving as mentors were provided with a cognitive behavioral therapy manual created by the researcher and asked to read it carefully.
(g) The nurse serving as the mentor conducted cognitive behavioral therapy on the mentee based on the manual.
(h) Each group consisted of two mentors and five mentees. During the session, only the mentee and mentor were involved; researchers and nursing department staff were not involved.
(i) While coordinating with researchers and the hospital’s nursing department, we were able to provide support should any problems arise.

### 2.4. Statistical Analysis

One-factor analysis of variance was performed on the above measures at pre-intervention, post-intervention, and 3 months follow-up, with statistical significance set at *p* ≤ 0.05. The multiple comparison procedure was performed in Tukey’s honestly significant difference test.

### 2.5. Ethical Considerations

This research was conducted in accordance with the Ethical principles of the revised Helsinki Declaration. This study was approved by the University of Hyogo University Ethics Review Committee (No. 15006). When conducting the study, a written and verbal research request was submitted to the administrator of the facility where the research was conducted, after which a written and verbal explanation was provided to the participants. Participants who provided written informed consent were included in the present study. In the written statement to the participants, they were informed about the voluntary nature of participation, the freedom to discontinue participation without penalty, and the restricted use of their responses for the present study. Furthermore, they were informed that their data would be statistically processed using a code, ensuring the preservation of their privacy and confidentiality. This research was conducted in accordance with the Ethical principles of the revised Helsinki Declaration. Data were saved on a secure USB. The data retention period was 5 years.

## 3. Results

### 3.1. Participants’ Basic Attributes of Mentees

The mentees included 33 nurses (4 males and 29 females) in their first to third year of nursing. The age groups of 20–25 and 26–31 years included 28 and 5 participants, respectively. The number of nurses in their first, second, and third year of nursing work was 15, 11, and 7, respectively (Table 2).

### 3.2. Participants’ Characteristics of Mentors

Mentors included 35 nurses (4 males and 31 females). Concerning their highest education, 21 had completed nursing school, 3 had completed junior college, 8 had completed university, 1 had completed graduate school, and 2 had completed advanced studies. Currently, 12 are posted in hospital wards, 10 in surgical wards, 2 in outpatient departments, 4 in operating rooms, 2 in the ICU, and 4 in pediatrics. All the participants received support from a preceptor system when they started working as nurses (Table 3).

### 3.3. Effectiveness of the Program

A CBT program was implemented with 35 staff-trained nurses as mentors and 33 young nurses in their first to third year as mentees. The results revealed that the stressors were “total strain” (F [2,91] = 3.78, *p* < 0.05), “conflict with other nursing staff” (F [2,91] = 4.06, *p* < 0.05), “nursing role conflict” (F [2,91] = 4.02, *p* < 0.05), “qualitative workload” (F [2,91] = 4.93, *p* < 0.05), “quantitative workload” (F [2,91] = 6.29, *p* < 0.01), “conflict of patients” (F [2,91] = 3.92, *p* < 0.05), “problem avoidance” in irrational beliefs (F [2,91] = 3.35, *p* < 0.05), “escape-avoidance coping” (F [2,91] = 4.21, *p* < 0.05), “emotional exhaustion” in burnout (F [2,91] = 3.73, *p* < 0.05), and “want to change hospital departments” in the intention to quit (F [2,91] = 6.12, *p* < 0.05) (Table 4).

## 4. Discussion

Nursing turnover is a serious problem in today’s society. Burnout has been identified as a contributing factor to nurse turnover. Mentorship refers to the process in which experienced individuals share their experiences and provide support to the mentee, not as a mentor but rather as a sounding board. The previous literature has demonstrated that mentorship reduces stress and anxiety among new nurses and improves their adaptability in the workplace [34]. Additionally, mentorship can reduce nurse turnover [34,35]. Therefore, mentorship is an effective means of preventing burnout among nurses. It is also influential in improving the mentoring skills of mentors [36]. To the best of our knowledge, no previous study has examined the effectiveness of CBT provided through a mentoring program for nurses with clear outcomes, such as burnout or turnover intention. This study examined the effectiveness of a mentoring system based on a CBT program [22] for improving nurses’ mental health.

The results of this study indicate that the CBT intervention significantly reduced many stressors. Particularly, reductions were observed in total strain, conflict with other nursing staff, nursing role conflict, qualitative workload, quantitative workload, and conflict with patients, indicating the effectiveness of mentor-delivered CBT in reducing stress in nursing practice. According to a previous study on burnout among nurses, total strain was found to be an indicator of the sum of stress responses and was used to measure the degree of burnout. Higher total strain was associated with a higher risk of burnout [37].

Furthermore, significant reductions were observed in irrational beliefs, maladaptive coping, and burnout, suggesting an effect on the mentees’ psychological health. Particularly, reductions in “problem avoidance” due to irrational beliefs and “escape-avoidance” style of coping may have improved nurses’ approach toward negative emotions and problems. Problem avoidance due to irrational beliefs means that an individual holds strong irrational beliefs and stereotypes and they avoid rather than deal with real problems [38]. Avoidance of problems due to irrational beliefs is one of the most common causes of burnout. CBT has been shown to be effective in reducing problem avoidance due to irrational beliefs [39]. It involves identifying irrational beliefs and developing specific strategies to challenge them. These specific methods include examining beliefs, modifying beliefs based on objective evidence, and changing behaviors based on the modified beliefs [39].

Moreover, a significant decrease was observed in “want to switch hospitals or departments” concerning their intent to leave the job. This suggests that CBT based on the mentor system may have contributed to the improvement of the nurses’ work–life balance and work environment.

The present study introduced a mentor system and implemented a CBT program including mid-career nurses (mentors) who received training to provide the program to their mentees. The results showed that the program effectively reduced mentees’ stress, burnout, and turnover.

However, this study has several limitations. First, the number of samples is small. the effectiveness of the intervention was assessed over a short duration; long-term follow-up is required. Additionally, the study participants were limited to nurses working in three hospitals, making it difficult to generalize the findings to other facilities and backgrounds. Furthermore, it is possible that mentoring itself provided psychological comfort to the mentees, which may have reduced their stress without CBT. Additionally, there are limitations in the research design, namely the absence of a control group.

The previous literature has also noted that the effectiveness of mentoring programs is limited. For example, if the mentor does not have sufficient guidance skills or is not a good match for the mentee, it can conversely increase their stress [40]. Organizational support is also necessary for the effective implementation of the mentoring program [41].

Therefore, further research is required to build on the findings of the present study and verify its effectiveness in diverse nursing populations and healthcare settings. Additionally, studies on specific implementation methods and customization of the mentoring system and CBT must be conducted to create a more practical and effective model of care. As a future project, the authors plan to compare CBT implemented by therapists with CBT implemented by mentors to examine the differences in the effectiveness of the intervention.

When considering the implications for clinical practice, it is important to recognize that implementing mentoring programs with CBT can significantly impact patient care. By enhancing nurses’ mental well-being, such programs can improve patient outcomes and overall healthcare quality. Additionally, integrating these programs into nursing training and education can better prepare future nurses to manage stress and prevent burnout, ultimately benefiting both healthcare providers and patients.

Furthermore, from a research perspective, exploring the effectiveness and long-term effects of mentoring programs with CBT on nurse retention, job satisfaction, and patient care outcomes is crucial. Continued research in this area can provide valuable insights into best practices for promoting nurse well-being and addressing workforce challenges in healthcare settings.

## 5. Conclusions

Overall, the present study suggests that mentoring programs and CBT may contribute to improving the mental health of nurses, leading to an improved working environment and reduced turnover. In summary, a mentoring program using CBT has the following advantages: (1) young nurses receive CBT from mid-career nurses with whom they have good interpersonal relations, leading to improved mental health, (2) mentors have experienced young nurses’ suffering and can empathize with them, (3) the role of providing mental health support is established, (4) the mentor from a different department creates a sense of security for the mentee in terms of their privacy, and (5) it also improves the mental health of trained mid-career nurses. It is hoped that the development of specific intervention programs and measures to improve the mental health of the nursing workforce will continue.

## Figures and Tables

**Table 1:** Mentor intervention program for mentees.

	Psycho-Educational Interventions	Group Work	Homework
Session 1	An orientation for psycho-educational interventions on stress and burnout among nurses and related factors, counseling mindset, and counseling techniques	Group work on perceived stress in performing current duties and listening and empathic understanding.	Self-monitoring of stress scenes
Session 2	Overview of CBT, and cognitive restructuring and its relation to stress	Group work involving role-playing stressful situations and experiencing how cognitive restructuring can change one’s mood by changing one’s thoughts.	Five column methods.
Session 3	Problem-solving skills training	Group work on problem-solving techniques	Practice of Problem-solving skills training

**Table 2:** Participants’ basic attributes of mentees.

		N	%
Gender	Males	4	12.1
	Females	29	87.9
Age	20–25	28	84.8
	26–31	5	15.2
Years of service	1 year	15	45.5
	2 years	11	33.3
	3 years	7	21.2
Educational background	Vocational school	11	33.3
	Junior college	3	9.1
	University	18	54.5
	Advanced course	1	3
Department	General wards	12	36.4
	Surgical ward	12	9.1
	Obstetrics and Gynecology ward	3	9.1
	Pediatric ward	4	12.1
	Operating room	2	6.1
Marital status	Married	1	3
	Unmarried	32	97

**Table 3:** Participants’ characteristics of mentors.

		n	%			n	%
Gender	Males	4	8	Qualification	Nurses	34	100
	Females	31	65		Public health nurses	6	13
Department	Medical wards	12	25		Midwife	3	6
	Surgical wards	10	21				
	Outpatient department	2	4	Work formats	Day shift	8	17
	Operating room	4	8		Day shift and duty	4	8
	Intensive care units	2	4		3 shifts	12	25
	Pediatric	4	8		2 shifts	10	21
Educational background	Vocational school	21	44		Night shift	0	0
	Junior college	3	6				
	University	8	17	The experience of preceptorship (guidance for newcomers)	Yes	34	100
	Graduate school	1	2		None	0	0
	Advanced course	2	4				

**Table 4:** Effects of cognitive behavioral therapy on mentee scales.

		Pretest		Posttest		Follow-Up		F	P	η^2^
		M	SD	M	SD	M	SD			
NJSS	The total strain	70.84	12.49	68.79	14.41	61.79	14.74	3.78	0.03	0.08
	Nursing role conflict	14.66	2.54	14.52	2.94	13.21	3.23	4.02	0.05	0.05
	Qualitative workload	16.19	2.81	15.93	2.94	14.21	3.70	6.29	0.01	0.07
	Quantitative workload	17.69	3.06	17.14	3.03	15.88	3.68	4.93	0.03	0.05
	Conflict with patients	5.94	1.72	5.38	1.74	4.70	1.90	3.92	0.02	0.08
	Conflict with other nursing staff	14.31	2.44	14.17	2.99	12.85	3.32	4.06	0.05	0.05
JIBT-20	The total of irrational belief	39.25	6.31	38.14	7.45	36.39	5.73	3.14	0.08	0.03
	Problem avoidance	12.38	2.66	11.10	3.06	10.55	3.00	3.35	0.04	0.07
	Helplessness	12.13	2.66	12.17	3.12	11.73	2.17	0.27	0.76	0.01
	Dependence	14.75	3.01	14.86	3.24	14.12	2.90	0.55	0.58	0.01
ATQ-R	Negative evaluation of the future	14.72	4.90	14.00	4.93	14.39	4.67	0.17	0.85	0.00
	Self-blame	16.44	4.20	16.86	4.25	16.50	4.14	0.09	0.91	0.00
	Positive thinking	17.38	4.17	17.21	4.31	16.55	4.51	0.33	0.72	0.01
Coping	Problem-focused coping	8.13	3.23	8.10	2.97	7.45	3.09	0.49	0.62	0.01
	Emotion-focused coping	5.66	2.31	5.34	1.90	4.91	1.94	1.08	0.34	0.02
	Escape-avoidance coping	11.00	3.42	9.59	2.85	9.00	3.50	4.21	0.04	0.05
MBI	Emotional exhaustion	21.38	3.32	20.45	4.01	18.70	4.59	3.73	0.03	0.08
	Depersonalization	12.50	4.79	12.28	4.52	10.97	4.52	1.04	0.36	0.02
	Personal accomplishment	12.77	3.74	12.86	3.64	13.15	3.61	0.09	0.91	0.00
Intention to leave the job	Wants to quit working as a nurse	3.34	1.29	3.24	1.15	2.85	1.39	2.41	0.12	0.03
	Wants to switch hospitals	3.06	1.37	2.34	1.29	2.24	1.35	6.12	0.02	0.07
	Wants to continue working as nurse	3.38	1.01	2.93	1.10	3.18	1.24	1.20	0.31	0.03

NJSS, JIBT-20: Only sub-items significant for burnout in the Ohue (2011) survey are used.

## Data Availability

The de-identified data underlying the results presented in this study are available upon request to the corresponding author.

## Public Involvement Statement

No public involvement in any aspect of this research.

## Guidelines and Standards Statement

Data are reported here according to the Strengthening the Reporting of Observational studies in Epidemiology (STROBE) statements to improve truthfulness and ensure clarity. “https://www.equator-network.org/reporting-guidelines/strobe/” (accessed on 28 August 2023).
